# The impact of adverse events on health-related quality of life among patients receiving treatment for drug-resistant tuberculosis in Johannesburg, South Africa

**DOI:** 10.1186/s12955-019-1155-4

**Published:** 2019-05-31

**Authors:** Tembeka Sineke, Denise Evans, Kathryn Schnippel, Heleen van Aswegen, Rebecca Berhanu, Nozipho Musakwa, Elisabet Lönnmark, Lawrence Long, Sydney Rosen

**Affiliations:** 1 Health Economics and Epidemiology Research Office, Department of Internal Medicine, School of Clinical Medicine, Faculty of Health Sciences, University of the Witwatersrand, Johannesburg, South Africa; 2 Health Economics Unit, Faculty of Health Sciences, School of Public Health and Family Medicine, Cape Town, South Africa; 3 Department of Physiotherapy, School of Therapeutic Sciences, Faculty of Health Sciences, University of the Witwatersrand, Johannesburg, South Africa; 4 Department of Global Health, Boston University School of Public Health, Boston, MA USA; 5 Department of Infectious Diseases, Sahlgrenska University Hospital, Gothenburg, Sweden

**Keywords:** Health-related quality of life (HRQoL), HIV/AIDS, DR-TB, Adverse events, SF-36, Mental health component summary scores, Physical health component summary scores

## Abstract

**Background:**

Adverse events (AEs) are common during treatment of drug-resistant tuberculosis (DR-TB). Little is known about the health-related quality of life (HRQoL) of patients receiving treatment for DR-TB or the effect of AEs on HRQoL.

**Methods:**

We conducted a cross-sectional study among adult patients with laboratory-confirmed rifampicin resistant tuberculosis (TB) on DR-TB treatment at a public-sector outpatient DR-TB clinic in Johannesburg, South Africa between 02/2015–01/2018. Data on HRQoL using the Medical Outcomes Short Form-36 (SF-36) questionnaire and self-reported AEs were collected by trained interviewers through face-to-face interviews. We report averages for the eight major domains and mental (MCS) and physical health (PCS) component summary scores, stratified by whether AEs were reported in the last four weeks. For comparative purposes, we enrolled two other patient groups and included data on a separate group of healthy adults.

**Results:**

We enrolled 149 DR-TB patients (median age 36 years IQR 29–43, 55% male, 77.9% HIV-positive, 81% on ART, 61.8% on a standard long-course regimen and 44.3% on DR-TB treatment for less than 6 months). 58/149 (38.9%) patients reported a total of 122 AEs in the preceding 4 weeks, of these the most common were joint pain (*n* = 22), peripheral neuropathy (*n* = 16), hearing loss (*n* = 15), nausea and vomiting (*n* = 12) and dizziness or vertigo (*n* = 11). SF-36 domains and summary scores (MCS and PCS) were lower in those who reported an AE compared to those who did not, and both were lower than healthy adults. Compared to those who did not report an AE, patients who reported AEs were more likely to have a low MCS (aRR 2.24 95% CI 1.53–3.27) and PCS (aRR 1.52 95% CI 1.07–2.18) summary score. HRQoL was lower among those on DR-TB treatment for 6 months or less.

**Conclusion:**

Results show that DR-TB had a substantial impact on patients’ quality of life, but that AEs during the early months on treatment may be responsible for reducing HRQoL even further. Our findings highlight the negative effects of injectable agents on HRQoL. Patients require an integrative patient-centered approach to deal with DR-TB and HIV and the potential overlapping toxicities which may be worsened by concurrent treatment.

**Electronic supplementary material:**

The online version of this article (10.1186/s12955-019-1155-4) contains supplementary material, which is available to authorized users.

## Background

South Africa bears a disproportionate share of the world’s epidemic of drug-resistant tuberculosis (DR-TB) [1], with an estimated 15,986 laboratory-confirmed rifampicin-resistant tuberculosis and multi-drug resistant (RR/MDR-TB) cases reported in 2017. Of these, 10,259 (64.2%) started appropriate DR-TB treatment [1]. The treatment for RR/MDR-TB takes much longer and is more complex and often more toxic than the treatment used for drug-susceptible TB (DS-TB) and is comprised of agents that are associated with the occurrence of serious adverse events (AE) [2, 3]. AEs range from ones that temporarily reduce quality of life (e.g. abdominal pain, rash, nausea, vomiting) to those that cause long-term disability (e.g. irreversible hearing loss) or are potentially life threatening (e.g. renal failure, psychosis, seizures) [3–6]. Studies have shown that up to 64% of patients require discontinuation of one or more drugs from their DR-TB treatment due to severe AEs [5, 7–9], which are particularly common during the intensive phase of therapy [6]. AEs have been shown to affect treatment adherence and are associated with poorer treatment outcomes, more specifically retention in care [2].

Tuberculosis has substantially adverse impacts on patient’s quality of life [10, 11]. Although the severity and extent of AEs relating to DR-TB treatment are well documented, little is known about health-related quality of life (HRQoL) of patients receiving treatment for DR-TB or the effect of AEs on HRQoL [9, 10, 12]. The evidence that is available shows that some AEs severely impact and lower patients’ HRQoL [10, 13–15] and that there is an association between quality of life and adherence to therapeutic recommendations (e.g. appointment keeping or adherence to medication) [2, 16].

Understanding patient experiences of AEs and how AEs affect their quality of life and their perception of care are important in creating treatment guidelines that better serve patients. Providing care that is responsive to the individual patient (i.e. an integrative patient-centered approach aimed at alleviating illness, suffering and death of individuals due to TB) may improve treatment outcomes and contribute towards meeting the United Nations Sustainable Development Goal 3 (SDG 3) target and support the End TB strategy of the World Health Organization (WHO) [17, 18].

To date, no published study has evaluated the association between the reporting of AEs and patients’ HRQoL scores during MDR-TB treatment, in a setting with high rates of HIV co-infection and antiretroviral therapy (ART) coverage. In this cross-sectional study, we describe the HRQoL of DR-TB patients in Johannesburg, South Africa, stratified by self-reported AEs in the past 4 weeks. We also present HRQoL of DR-TB patients disaggregated by (i) duration of DR-TB treatment, (ii) type of DR-TB regimen and (iii) HIV status. We compare the SF-36 domains to HIV positive patients who were on ART for at least 6 months either with or without DS-TB (within the past 12 months) and healthy adults (no self-reported chronic or infectious diseases; i.e. HIV and TB uninfected).

**Fig. 1:**
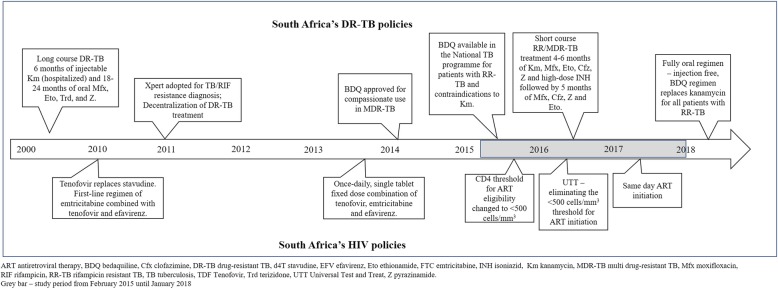
South Africa's DR-TB policies over the years

Over the course of our study, there have been substantial changes to the treatment guidelines for both DR-TB and HIV/AIDS in South Africa (Fig. 1). While South Africa has opted for all oral regimens for RR/MDR-TB by replacing injectables with bedaquiline and linezolid [19], the WHO has taken a more conservative approach and proposed two options: an all-oral, long, 18–20-month regimen or a shorter, injectable-based, 9–12-month regimen [20]. These changes are likely to affect AE profiles, but additional evidence on the negative impact of injectable agents on HRQoL may help countries decide to adopt oral or injectable regimens for RR/MDR-TB treatment in their national programs.

## Methods

We conducted a cross-sectional study among adult patients on DR-TB treatment at a public sector, decentralized outpatient treatment site in Johannesburg, South Africa. For this cross-sectional study, a census of patients from a larger on-going, prospective observational cohort study of RR/MDR-TB patients at the same facility (Wits HREC protocol M130205) who met the eligibility criteria were selected. This cohort has been described in detail elsewhere [21].

To better understand the HRQoL scales we enrolled two comparison groups from two primary health facilities (PHCs) and a hospital-based HIV clinic in Johannesburg, South Africa. The facilities were purposely selected because they treat specific patient types (e.g. HIV-positive patients on ART with or without DS-TB) in whom we were interested and because they are located in the same catchment area (within < 5 km of one another; in City of Johannesburg Region G) as the decentralized outpatient DR-TB treatment facility. We also included data from a published study on a separate group of healthy adults [22], for comparative purposes.

### Study population

To describe the effect of AEs on HRQoL, we included adults (≥18 years) with documented laboratory confirmed RR/MDR-TB, who were enrolled in the larger prospective cohort study after 1 March 2013 and returned to the clinic for DR-TB treatment between 1 February 2015 and 1 January 2018. Interviewers reviewed the DR-TB register at the healthcare facility to identify potential participants and recorded their upcoming visit date. When patients returned to the clinic interviewers identified and approached patients, informed them of the study, confirmed eligibility and obtained written informed consent.

For the comparison groups, we enrolled a sequential sample of patients as they presented at the facilities between January 2016 and January 2018. We included adult patients (≥18 years) who were HIV-positive and on ART for at least 6 months, either with or without DS-TB (within the past 12 months).

### Treatment regimens

There were several RR/MDR-TB treatment regimen and diagnostic guidelines in effect over the course of this study (Fig. 1). In 2011, South Africa introduced Xpert MTB/RIF for the diagnosis of TB and rifampicin (RIF) resistance. At the same time the National TB program adopted a policy of decentralized, outpatient treatment for DR-TB [23]. In 2013, the standard long-course RR/MDR-TB regimen remained in use. This consisted of 6 months of injectable kanamycin and 18–24 months of oral moxifloxacin, ethionamide, terizidone, and pyrazinamide. In October 2014, the South African Medicines Control Council regulatory authority approved the use of bedaquiline (BDQ) for compassionate use in MDR-TB. In March 2015, BDQ became available in the South African National TB Programme (NTP) [24]. BDQ was introduced as a substitute for kanamycin in patients with either contraindication to receiving an aminoglycoside (baseline hearing loss or renal dysfunction) or for those who developed an AE to standard treatment. In addition, patients with MDR-TB and both the *inhA* and *katG* isoniazid drug resistance mutations were also given BDQ in an injection-free regimen [25]. In May 2016, the SA NTP implemented short course RR/MDR-TB treatment which consisted of 6 months intensive phase of kanamycin, moxifloxacin, ethionamide, clofazimine, pyrazinamide, and high-dose isoniazid followed by 5 months of moxifloxacin, clofazimine, pyrazinamide, and ethambutol.

For HIV and ART, South Africa began initiating patients on a once-daily, single tablet fixed dose combination of tenofovir, emtricitabine and efavirenz after April 2013. Tenofovir replaced stavudine, a drug known to cause serious side effects, in a triple combination regimen [26]. In September 2016, South Africa implemented Universal Test & Treat (UTT) guidelines, eliminating the CD4 count < 500 cells/mm^3^ threshold for ART initiation and then in July 2017, in line with WHO recommendations, adopted same day ART initiation for clinically stable patients [27].

### Data collection

Data were collected by trained interviewers through face-to-face interviews using the Medical Outcomes Short Form-36 (SF-36) [28–30] (https://www.rand.org/health-care/surveys_tools/mos/36-item-short-form/scoring.html) questionnaire for HRQoL and the Patient-Reported Adverse Drug Event Questionnaire which is a validated tool to assess patient-reported AE [11, 31]. The SF-36 questionnaire has been validated for consistency and reliability in different settings for a multitude of health conditions including TB [12, 32, 33]. Among those who reported hearing loss, we used the Hearing Handicap Inventory for the Elderly Screening Version (HHIE-S) to assess how individuals perceived the social and emotional effects of hearing loss (i.e. no, mild-moderate or severe handicap) [11, 12, 31–34]. For those with hearing loss, persons accompanying the patient to the clinic (e.g. family member) helped to interpret the questions for the patient.

### Sources of data

All data collected during the interviews were collected on paper forms and later captured into REDCap [35] by study staff for data cleaning and analysis. Patient demographics, including gender, age, education, HIV status, DR-TB treatment start date, treatment regimen, and disease classification were obtained from the facility’s electronic patient record database. Survey responses were linked to patient demographics using the unique study ID which was assigned at enrolment in the prospective observational cohort study and a single analytic dataset was created.

For the comparison groups, SF-36 data were captured directly into REDCap using a Samsung Galaxy tablet. Interviewers also collected basic demographic and clinical information from the patient’s file. Data from a published report, which enrolled 40 healthy adults in Johannesburg (i.e. also in City of Johannesburg Region G), were used as a separate reference group [22].

### Study variables

For the analysis, we scored the SF-36 according to the scoring guideline for this instrument [28–30] (Additional file 1: Table S1). The eight domains (i.e. physical functioning, bodily pain, role limitations due to physical health problems, role limitations due to personal or emotional problems, emotional well-being, social functioning, energy/fatigue, and general health perceptions) were aggregated into two summary measures: the physical (PCS) and mental (MCS) component summary scores.

The Patient-Reported Adverse Drug Event Questionnaire [11, 31] lists over 400 possible symptoms that a patient could experience in the past 4 weeks, many of which are not related to DR-TB treatment (e.g. greasy skin, blushing, sneezing, goosebumps etc.). For the analysis, all symptoms or complaints reported were reviewed by a clinician (RB) and those unrelated to DR-TB treatment were excluded. The remaining symptoms were grouped into common AEs associated with RR/MDR-TB medication (See Additional file 3: Table S3 for events included). Patients were classified as having an AE (any grade) associated with RR/MDR-TB medication if they reported any of the included symptoms/AEs to the study interviewer in the past 4 weeks.

Patient characteristics recorded at treatment initiation included gender (male, female), age (18–35, ≥35 years), education (secondary school and higher, primary school and less) and employment status. Employment was classified as either unemployed (including students or retired) or employed (including self-employed or casual employment). We classified the resistance pattern according to the diagnostic method used: rifampicin (RIF) resistant by Xpert MTB/RIF (Cepheid, USA; RR-TB diagnosed by Xpert MTB/RIF with unknown or pending sensitivity to isoniazid or second-line TB drugs), MDR-TB (RIF and isoniazid resistant), or RIF mono-resistant (mono- or poly-resistant resistance to RIF alone or RIF plus another first-line drug other than isoniazid, confirmed by line probe assay or DST).

HIV status was obtained from the facility’s electronic patient record database and categorized as HIV-negative, HIV-positive on ART, HIV-positive not on ART, or HIV status unknown. Additional patient information collected at treatment initiation included diabetes (no, yes, missing/unknown), weight (< 50 kg, ≥50 kg, missing), referring facility (outpatient, inpatient), patient category (new, previously treated), TB type (pulmonary, extra pulmonary) and smear microscopy (negative, positive, unknown). For defining anaemia, haemoglobin (Hb) was adjusted downward by 0.65 g/dL to account for elevation above sea level in Johannesburg [36]. Anaemia was categorized according to WHO guidelines as none (Hb ≥12 g/dL for non-pregnant women and Hb ≥13 g/dL for men), mild (Hb 11–11.9 g/dL for non-pregnant women and 11–12.9 g/dL for men), moderate (8–10.9 g/dL) and severe (Hb < 8 g/dL) [37].

Duration of ART and duration of DR-TB treatment were calculated from the treatment start date until the date of the interview, and were categorized as ≤6 vs. > 6 months. DR-TB treatment was categorized as either currently (i.e. date of the interview) receiving a standard or individualized (i.e. BDQ substituted for kanamycin) long- or short-course regimen. Patients who were categorized as being on an injection-free, BDQ-containing regimen, were either started on BDQ at baseline or were switched to BDQ due to an incident AE such as hearing loss or renal failure.

### Statistical analysis

The primary outcomes of the analysis were low MCS or low PCS summary score which were determined using the median as a cut-off. Patients with a norm-based MCS or PCS summary score below the median cut-off were defined as having a low MCS or PCS summary score while patients with a summary score above or equal to the median cut-off were defined as having a normal-high MCS or PCS summary score.

Patient demographic and clinical characteristics at the start of DR-TB treatment were summarized using frequencies for categorical variables, means with standard deviation for normally distributed data or median and interquartile range (IQR) for not normally distributed data. We present patient demographics and clinical characteristics, stratified by whether the patients reported an AE associated with DR-TB treatment in the past 4 weeks. We present the mean, standard deviation, and Cronbach’alpha for the eight domains (normal additive approach and norm-based approach) and the MCS and PCS summary scores, again stratified by whether the patients reported an AE in the past 4 weeks or not. For reliability, a Cronbach’ alpha value of > 0.80 was used to define good internal consistency of the SF-36 domains (Additional file 1: Table S1). To compare domains and summary scores for those who reported an AE versus those who did not, we used Wilcoxon rank sum or Kruskal-Wallis test for non-parametric data and the student t test for parametric or normally distributed data. We further stratified the summary scores by HIV status, duration of DR-TB treatment, and DR-TB regimen and present this alongside the summary scores for the comparison groups.

We identified patient characteristics associated with a low MCS and low PCS summary score respectively using a Poisson regression model to estimate the relative risk (RR) and 95% confidence interval. Variables in the univariate model that were significant at the 0.2 level along with variables known to be associated with the outcome of interest (e.g. age, gender, resistance pattern etc.) and potential confounders (i.e. variable changes the estimate by > 10%) were included in the multivariate regression model. The univariate and multivariate (adjusted) results are presented.

All analyses were carried out using STATA version 13 (STATA Corp, Texas, USA) and SAS version 9.3 (SAS Institute Inc., Cary, NC, USA). This study and the analysis of anonymized data was approved by the Human Research Ethics Committee (Medical) of the University of the Witwatersrand (Wits HREC M141188). All participants provided written informed consent to participate in the study.

## Results

### Demographic and clinical characteristics

A total of 149 DR-TB patients were enrolled (Table 1). Patients had a median age of 36 years (IQR 29–43), 55.0% were male, and 42.3% were employed. Close to half (45.0%) of patients were classified as RIF mono-resistant (isoniazid sensitive), 25.5% had RIF resistance diagnosed by Xpert MTB/RIF with no further susceptibility testing and the remaining 21.5% were classified as MDR-TB. More than half (55.7%) of the patients had been on DR-TB treatment for more than 6 months. The majority (77.9%) were HIV positive; among these 81% were on ART, with 71.3% on ART for more than 6 months.

**Table 1:** Characteristics of patients by experience with adverse event during DR-TB treatment (*n* = 149)

	Adverse event not reported (*n* = 91)	Patient-reported adverse event (*n* = 58)	Total (n = 149)
	n (%)	n (%)	n (%)
Sex			
Male	47 (51.6%)	35 (60.3%)	82 (55.0%)
Female	44 (48.4%)	23 (39.7%)	67 (45.0%)
Age at treatment initiation (years)			
Median (IQR)	36 (29–44)	35 (29–42)	36 (29–43)
18–35	45 (49.5%)	29 (50%)	74 (49.7%)
≥ 35	46 (50.5%)	29 (50%)	75 (50.3%)
Education			
Secondary school and higher	86 (94.5%)	49 (84.5%)	135 (90.6%)
Primary school or less	5 (5.5%)	9 (15.5%)	14 (9.4%)
Employment Status			
Unemployed	47 (51.6%)	38 (65.5%)	85 (57.0%)
Employed	43 (47.3%)	20 (34.5%)	63 (42.3%)
Missing/unknown	1 (1.1%)	0 (0%)	1 (0.7%)
Resistance Pattern			
MDR-TB (RIF and INH resistant)	22 (24.2%)	10 (17.2%)	32 (21.5%)
RIF resistant by Xpert MTB/RIF	22 (24.2%)	16 (27.6%)	38 (25.5%)
RIF mono-resistant (INH sensitive)	39 (42.9%)	28 (48.3%)	67 (45.0%)
Missing	8 (8.8%)	4 (6.9%)	12 (8.1%)
HIV Status			
HIV negative	18 (19.8%)	9 (15.5%)	27 (18.1%)
HIV positive	69 (75.8%)	47 (81.0%)	116 (77.9%)
HIV positive and on ART	60 (87.0%)	34 (72.3%)	94 (81.0%)
HIV positive not on ART	9 (13.0%)	13 (36.2%)	22 (19.0%)
Unknown	4 (4.4%)	2 (3.5%)	6 (4.0%)
Baseline CD4 (cells/mm^3^)^#^			
< 50	23 (31.5%)	9 (18.4%)	32 (26.2%)
51–250	21 (27.8%)	22 (44.9%)	43 (35.3%)
> 250	22 (30.1%)	14 (28.6%)	36 (29.5%)
Missing	7 (69.6%)	4 (8.2%)	11 (9.0%)
DR-TB regimen			
Standard long-course^a^	61 (67.0%)	31 (53.4%)	92 (61.8%)
Individualized long-course^b^	17 (18.7%)	16 (27.6%)	33 (22.1%)
Standard short-course^c^	9 (9.9%)	6 (10.3%)	15 (10.1%)
Individualized short-course^d^	4 (4.4%)	5 (8.6%)	9 (6.0%)
Duration of DR-TB treatment (months)			
≤ 6 months	37 (40.7%)	29 (50.0%)	66 (44.3%)
> 6 months	54 (59.3%)	29 (50.0%)	83 (55.7%)
Duration of ART (months)^&^			
≤ 6 months	9 (15.0%)	5 (14.7%)	14 (14.9%)
> 6 months	44 (73.3%)	23 (67.6%)	67 (71.3%)
Missing	7 (11.7%)	6 (17.6%)	13 (13.8%)
Diabetes			
No	67 (73.6%)	42 (72.4%)	109 (73.2%)
Yes	4 (4.4%)	2 (3.5%)	6 (4.0%)
Missing	20 (22.0%)	14 (24.1%)	34 (22.8%)
Anaemia			
None or mild (Hb ≥11.0 g/dL)	46 (50.5%)	18 (31.0%)	64 (43%)
Moderate (8–10.9 g/dL) or severe (< 8 g/dL)	13 (14.3%)	13 (22.4%)	26 (17.4%)
Missing	32 (35.2%)	27 (46.6%)	59 (39.6%)
Weight at treatment initiation (kg)			
< 50 kg	19 (20.9%)	18 (31.0%)	37 (24.8%)
≥ 50 kg	65 (71.4%)	38 (65.5%)	103 (69.1%)
Missing	7 (7.7%)	2 (3.4%)	9 (6%)
Referring Facility			
Outpatient	62 (68.1%)	38 (65.5%)	100 (67.1%)
Inpatient	29 (31.9%)	20 (34.5%)	49 (32.9%)
Patient Category			
New	52 (57.1%)	38 (65.5%)	90 (60.4%)
Previously treated	23 (25.3%)	13 (22.4%)	36 (24.2%)
Missing	16 (17.6%)	7 (12.1%)	23 (15.4%)
TB Type			
PTB and EPTB or EPTB only	14 (15.4%)	10 (17.2%)	24 (16.1%)
PTB and not reported	77 (84.6%)	48 (82.8%)	125 (83.9%)
Smear Microscopy			
Negative	57 (62.6%)	42 (72.4%)	99 (66.4%)
Positive	17 (18.7%)	9 (15.5%)	26 (17.4%)
Unknown	17 (18.7%)	7 (12.1%)	24 (16.1%)

*PTB* pulmonary tuberculosis, *EPTB* extra pulmonary tuberculosis, *DR-TB* drug-resistant TB, *MDR-TB* multi-drug resistant TB, *RR-TB* rifampicin-resistant tuberculosis, *RIF* rifampicin, *INH* isoniazid, *Hb* hemoglobin

# Among patients who are HIV positive (*n* = 116)

& Among patients who are HIV positive and on ART (*n* = 94)

^a^ Standard long-course = 6 months of injectable kanamycin and 18–24 months of oral moxifloxacin, ethionamide, terizidone, and pyrazinamide

^b^ Individualized long-course = bedaquiline was introduced as a substitute for kanamycin in the standard long-course regimen (either at start of DR-TB or switched during treatment due to an incident adverse event)

^c^ Standard short-course = 4 to 6-month intensive phase of kanamycin, moxifloxacin, ethionamide, clofazimine, pyrazinamide and high-dose isoniazid followed by 5 months of moxifloxacin, clofazimine, pyrazinamide and ethambutol

^d^ Individualized short-course = bedaquiline was introduced as a substitute for kanamycin in the standard short-course regimen (either at start of DR-TB or switched during treatment due to an incident adverse event)

Overall, 38.9% (58/149) of patients self-reported to the interviewer a total of 122 AEs in the preceding 4 weeks, of which joint pain (*n* = 22), peripheral neuropathy (*n* = 16), hearing loss (*n* = 15), nausea and vomiting (*n* = 12), and dizziness or vertigo (*n* = 11) were the most common (Additional file 2: Table S2). Baseline characteristics of those who reported an AE versus those who did not were largely similar, though patients who reported an AE were more likely to have moderate or severe anemia (from laboratory results) (< 11 g/dL vs. ≥12 g/dL; RR 1.78 95% CI 1.03–3.08) and were less likely to have more than a secondary school education (RR 0.56 95% CI 0.36–0.89) (Table 1).

For the comparison groups, we enrolled 18 patients who were HIV-positive on ART with DS-TB (median age 37 IQR 32–47; 44.4% male) and 50 patients who were HIV positive on ART (no TB) (median age 44 IQR 35–48; 40.0% male). For the published (healthy) comparison group, the mean age of this group was 29.5 (SD ±8.5) years with 39 males and one female [22].

### Comparison of SF-36 scores and MCS and PCS component summary scores

Patients on DR-TB treatment who reported an AE had lower domains compared to patients who did not. Physical, emotional and social function were noticeably reduced among patients who reported an AE compared to those who did not. When comparing the MCS and PCS summary score, patients who reported an AE had lower summary scores than those who did not (MCS 32.1 vs. 42.2; PCS 46.5 vs. 52.8). Results were consistent across both the normal additive approach (not standardized) and the norm-based approach (standardized) (Table 2).

**Table 2:** Comparison of SF-36 health domain scales (normal and norm-based) between DR-TB patients who reported an adverse event in the last four weeks and those who did not (*n* = 149)

		Adverse event not reported (n = 91)			Patient-reported adverse event (n = 58)			
SF-36 scale^a^ (Normal scale)	Items	Mean	SD	Alpha*	Mean	SD	Alpha*	*P* value**
Physical functioning	10	89.0	16.9	0.910	73.0	24.8	0.869	0.0001
Role functioning/physical	4	68.7	43.2	0.906	29.7	43.3	0.849	0.0001
Role functioning/emotional	3	64.8	46.7	0.909	22.9	39.1	0.866	0.0001
Energy/fatigue	4	58.3	16.3	0.903	48.3	17.1	0.861	0.006
Emotional well-being	5	65.3	17.8	0.902	54.8	17.1	0.851	0.005
Social functioning	2	78.6	22.8	0.908	59.5	16.6	0.859	< 0.0001
Pain	2	78.5	20.9	0.906	63.4	12.0	0.86	0.0001
General health	5	66.6	15.1	0.914	59.3	13.8	0.869	0.003
SF-36 scale^a^ (Norm-based scale)								
Physical functioning	10	52.4	7.1	0.911	45.7	14.9	0.869	0.0001
Role functioning/physical	4	44.6	16.9	0.907	29.3	17.7	0.849	0.0001
Role functioning/emotional	3	39.4	21.8	0.91	19.9	18.2	0.866	0.0001
Energy/fatigue	4	50.0	8.1	0.904	44.9	8.5	0.861	0.0005
Emotional well-being	5	44.5	10.0	0.903	38.6	8.6	0.851	0.007
Social functioning	2	47.5	9.9	0.909	39.2	8.1	0.859	0.0001
Pain	2	53.0	8.8	0.907	46.2	10.6	0.86	0.0001
General health	5	48.0	7.2	0.914	44.5	6.6	0.869	0.003
Mental health component summary (MCS)		42.2	13.0	n/a	32.1	9.9	n/a	< 0.001
Physical health component summary (PCS)		52.8	8.2	n/a	46.5	9.1	n/a	< 0.0001

^a^ using the 36-Item Medical Outcomes Short Form Health Survey (SF-36)

*For the Alpha, Cronbach’ alpha value of >0.80 was used to define good internal consistency of the SF-36 domains

**For the *P* value, this was to indicate the difference between means for patients with AE vs those without. 0.05 was a cutoff for significance

For all DR-TB patients, those who reported an AE and those who did not, the MCS was lower than the PCS (Table 3). DR-TB patients co-infected with HIV, but not on ART, had a lower MCS and PCS summary score than did co-infected patients who were on ART or patients who were HIV negative (MCS 34.5 vs. 38.7 or 41.4; PCS 46.7 vs. 51.2 or 50.9). DR-TB patients who had been on treatment for 6 months or less had a lower MCS and PCS summary score than patients who had been on treatment for more than 6 months (MCS 31.9 vs. 42.9; PCS 48.5 vs. 52.6).

**Table 3:** MCS and PCS summary scores for DR-TB patients and those enrolled in the comparison groups

	Mental component summary (MCS)		Physical component summary (PCS)	
SF-36 scale^$^ (Norm-based scale)	Mean	SD	Mean	SD
DR-TB (all patients; n = 149)	38.3	12.9	50.4	9.1
Patient-reported adverse event				
No adverse event	42.2	13.0	52.8	8.2
Patient-reported adverse event	32.1	9.9	46.5	9.1
HIV status				
HIV negative (*n* = 27)	41.4	11.6	50.9	8.7
HIV positive on ART (n = 94)	38.7	12.9	51.2	8.5
HIV positive not on ART (n = 22)	34.5	13.9	46.7	10.7
HIV status unknown (*n* = 6)	32.7	12.5	49.0	11.6
Duration of DR-TB treatment				
≤ 6 months (*n* = 66)	31.9	11.3	48.5	9.9
> 6 months (*n* = 83)	42.9	12.0	52.6	8.0
DR-TB regimen				
Standard regimen (long- and short-course) (*n* = 107)	37.9	13.4	51.1	8.9
Did not report an AE	41.4	13.6	53.2	8.2
Self-reported AE	31.4	10.4	47.1	8.8
Individualized regimen (long- and short-course) (*n* = 42)	39.3	11.6	48.7	9.4
Did not report an AE	45.1	10.8	51.7	8.3
Self-reported AE	33.4	9.3	45.6	9.7
Comparison groups				
HIV positive on ART with DS-TB (*n* = 18)	44.4	15.7	48.5	9.6
HIV positive on ART no TB (*n* = 50)	43.3	11.5	54.8	7.3
Healthy Adults^a^ (*n* = 40)	50.3	10.3	57.6	5.1

^a^From published data (van Aswegen et al., 2011)

^$^Using the 36-Item Medical Outcomes Short Form Health Survey (SF-36)

Among HIV-positive patients on ART, DR-TB patients had a lower MCS than did those with DS-TB and those without TB (38.7 vs. 44.4 and 43.3) while both DR-TB and DS-TB patients had a lower PCS summary score than patients without TB (51.2 and 48.5 vs. 54.8) (Table 3). From published data, healthy adults in Johannesburg have a MCS and PCS summary score of 50.3 and 57.6, respectively (Additional file 3: Table S3) [22].

### Adverse events and HRQoL by DR-TB regimen

Two-thirds of patients (61.8%) were on a standard long-course regimen, 10.1% were on a standard short-course regimen, and 28.1% had either switched to (*n* = 35/42) or initiated on an injection-free regimen (*n* = 7/42) (Table 1). Median time on BDQ at time of interview for those on BDQ was 3.1 months (IQR 0.7–6.3). A higher proportion of patients on a standard regimen (long- or short-course with injectables) experienced AEs than those on an individualized regimen (injection-free, BDQ-containing regimen) (40.2% vs. 34.4%). Patients on an individualized regimen were less likely to report an AE in the past 4 weeks, however the estimate lacked precision possibly because the numbers within each strata were quite small (RR 0.86 95% CI 0.51–1.45). Of the 15 patients that experienced hearing loss, two thirds were on a standard long-course regimen and the remaining one third were on an individualized long-course regimen (Additional file 2: Table S2). The MCS and PCS summary scores were similar among patients on a standard (i.e. containing an injectable agent; e.g. kanamycin) or an individualized regimen (i.e. injection-free regimen) (MCS 37.9 vs. 39.3; PCS 51.1 vs. 48.7). However, all patients that reported an AE, whether on a standard or individualized regimen, had a lower MCS and PCS than patients that did not report an AE (Table 3).

### Factors associated with low MCS or PCS component summary scores

Among DR-TB patients, we identified factors associated with having a low MCS score, as shown in Table 4. From the multivariate regression, DR-TB patients who reported an AE (aRR 2.24 95% CI 1.53–3.27) and those on DR-TB treatment for less than 6 months (≤6 vs. > 6 months; aRR 2.27 95% CI 1.53–3.35) were more likely to have a low MCS summary score. After adjusting for patient-reported AE, gender, age, resistance pattern and duration of DR-TB treatment, patients who were on a standard long- or short-course DR-TB regimen were more likely to have a low MCS summary score than those who either switched to or initiated on an injection-free regimen (aRR 1.49 95% CI 1.00–2.24).

**Table 4:** Demographic and clinical factors associated with having a low MCS or PCS component summary score

	Mental component summary score			Physical component summary score		
	N, % (*n* = 74)	Crude RR (95% CI)	Adjusted RR (95% CI)	N, % (*n* = 75)	Crude RR (95% CI)	Adjusted RR (95% CI)
Adverse event						
Adverse event not reported	31/91 (34.1%)	1.00	1.00	35/91 (38.5%)	1.00	1.00
Adverse event reported	43/58 (74.1%)	2.18 (1.57–3.01)	2.24 (1.53–3.27)	40/58 (69.0%)	1.79 (1.31–2.45)	1.52 (1.07–2.18)
Sex						
Male	39/82 (47.6%)	1.00	1.00	41/82 (46.1%)	1.00	1.00
Female	35/67 (52.2%)	1.10 (0.79–1.52)	1.23 (0.81–1.84)	34/67 (54.8%)	1.01 (0.74–1.40)	1.10 (0.71–1.68)
Age at treatment initiation (years)						
18–35	39/74 (52.7%)	1.00	1.00	37/74 (50.0%)	1.00	1.00
> 35+	35/75 (46.7%)	0.89 (0.64–1.23)	0.91 (0.62–1.34)	38/75 (50.7%)	1.01 (0.74–1.40)	0.86 (0.56–1.31)
Education						
Secondary school and higher	67/135 (49.6%)	1.00		71/135 (52.6%)	1.00	
Primary school or less	7/14 (50.0%)	1.01 (0.58–1.75)		4/14 (28.6%)	0.54 (0.23–1.27)	
Employment Status						
Unemployed	45/85 (52.9%)	1.00		45/85 (52.9%)	1.00	
Employed	28/63 (44.4%)	0.84 (0.60–1.18)		29/63 (44.4%)	0.87 (0.62–1.22)	
Resistance Pattern						
RIF resistant by Xpert MTB/RIF	21/38 (55.2%)	1.00	1.00	21/38 (55.2%)	1.00	1.00
RIF mono-resistant (INH sensitive)	30/67 (44.8%)	0.81 (0.55–1.20)	0.89 (0.55–1.45)	29/67 (43.3%)	0.78 (0.53–1.17)	0.88 (0.55–1.41)
MDR-TB (RIF and INH resistant)	14/32 (43.8%)	0.79 (0.49–1.29)	1.09 (0.67–1.78)	16/32 (50.0%)	0.90 (0.58–1.42)	1.15 (0.66–1.99)
Missing	9/12 (75.0%)	1.36 (0.88–2.09)	0.98 (0.59–1.64)	9/12 (75.0%)	1.36 (0.88–2.09)	1.26 (0.81–1.95)
HIV Status						
HIV negative	11/27 (40.7%)	1.00		13/27 (48.1%)	1.00	
HIV positive and on ART	45/94 (47.9%)	1.18 (0.71–1.94)		44/94 (46.8%)	0.97 (0.62–1.52)	
HIV positive not on ART	14/22 (63.6%)	1.56 (0.90–2.72)		15/22 (68.2%)	1.42 (0.87–2.30)	
Missing	4/6 (66.7%)	1.64 (0.79–3.39)		3/6 (50.0%)	1.04 (0.42–2.54)	
Baseline CD4^#^						
≤ 250 (*n* = 78)	39/78 (50.0%)	1.00		41/78 (52.6%)	1.00	1.00
> 250 (*n* = 31)	16/31 (51.6%)	1.03 (0.69–1.55)		12/31 (38.7%)	0.74 (0.45–1.21)	0.82 (0.45–1.50)
CD4 count unknown (n = 7)	4/7 (57.1%)	1.14 (0.58–2.56)		6/7 (85.7%)	1.63 (1.13–2.36)	1.04 (0.55–1.94)
Indications for DR-TB regimen						
Standard regimen (long- or short-course)	55/107 (51.4%)	1.14 (0.78–1.66)	1.49 (1.00–2.24)	54/107 (50.5%)	1.01 (0.71–1.44)	
Individualized regimen (injection free)	19/42 (45.2%)	1.0	1.0	21/42 (50.0%)	1.0	
Duration of DR-TB treatment (months)						
≤ 6 months	49/66 (74.2%)	2.46 (1.72–3.53)	2.27 (1.53–3.35)	44/66 (66.7%)	1.78 (1.29–2.48)	1.70 (1.11–2.61)
> 6 months	25/83 (30.1%)	1.0	1.0	31/83 (37.3%)	1.0	1.0
Duration of ART (months)^&^						
≤ 6 months (n = 14)	10/14 (71.4%)	1.91 (1.21–3.02)	1.46 (0.96–2.23)	7/14 (50.0%)	1.24 (0.68–2.27)	0.98 (0.51–1.89)
> 6 months (*n* = 67)	25/67 (31.3%)	1.0	1.0	27/67 (40.3%)	1.0	1.0
ART start date unknown (*n* = 13)	10/13 (76.9%)	2.06 (1.34–3.18)	1.58 (0.96–2.59)	10/13 (76.9%)	1.91 (1.26–2.90)	1.58 (0.96–2.60)
Anaemia						
None or mild (Hb ≥11.0 g/dL)	29/64 (45.3%)	1.00		31/64 (48.8%)	1.00	
Moderate (8–10.9 g/dL) or severe (< 8 g/dL)	14/26 (53.8%)	1.19 (0.76–1.86)		14/26 (53.8%)	1.11 (0.72–1.72)	
Missing	31/59 (52.5%)	1.16 (0.81–1.67)		30/59 (50.8%)	1.05 (0.73–1.50)	
Weight at diagnosis (kg)						
< 50 kg	52/103 (50.5%)	1.00		51/103 (49.5%)	1.00	
≥ 50 kg	17/37 (45.9%)	0.91 (0.61–1.36)		18/37 (48.6%)	0.98 (0.67–1.44)	
Missing	5/9 (55.6%)	1.10 (0.59–2.04)		6/9 (66.7%)	1.35 (0.81–2.23)	
Referring Facility						
Outpatient	47/100 (47.0%)	1.00		48/100 (48.0%)	1.00	
Inpatient	27/49 (55.1%)	1.17 (0.84–1.63)		27/49 (55.1%)	1.15 (0.83–1.59)	
Patient Category						
New	47/90 (52.2%)	1.00		47/90 (52.2%)	1.00	
Previously treated	13/36 (36.1%)	0.69 (0.43–1.12)		13/36 (36.1%)	0.69 (0.43–1.12)	
Missing	14/23 (60.9%)	1.17 (0.79–1.71)		15/23 (65.2%)	1.25 (0.87–1.79)	
TB Type						
PTB and EPTB or EPTB only	11/24 (45.8%)	1.00		12/24 (45.8%)	1.00	
PTB and not reported	63/125 (50.4%)	1.10 (0.69–1.76)		63/125 (50.4%)	1.01 (0.65–1.56)	
Smear Microscopy						
Negative	49/99 (49.5%)	1.00		48/99 (48.5%)	1.00	
Positive	10/26 (38.5%)	0.78 (0.46–1.32)		12/26 (46.2%)	0.95 (0.60–1.51)	
Missing	15/24 (62.5%)	1.26 (0.87–1.83)		15/24 (62.5%)	1.29 (0.89–1.87)	

*PTB* pulmonary tuberculosis, *EPTB* extra pulmonary tuberculosis, *DR-TB* drug-resistant TB, *MDR-TB* multi-drug resistant TB, *RR-TB* rifampicin-resistant tuberculosis, *RIF* rifampicin, *INH* isoniazid, *Hb* hemoglobin

# Among patients who are HIV positive (n = 116)

& Among patients who are HIV positive and on ART (n = 94)

^a^ Standard long-course = 6 months of injectable kanamycin and 18–24 months of oral moxifloxacin, ethionamide, terizidone, and pyrazinamide

^b^ Individualized long-course = bedaquiline was introduced as a substitute for kanamycin in the standard long-course regimen (either at start of DR-TB or switched during treatment due to an incident adverse event)

^c^ Standard short-course = 4 to 6-month intensive phase of kanamycin, moxifloxacin, ethionamide, clofazimine, pyrazinamide and high-dose isoniazid followed by 5 months of moxifloxacin, clofazimine, pyrazinamide and ethambutol

^d^ Individualized short-course = bedaquiline was introduced as a substitute for kanamycin in the standard short-course regimen (either at start of DR-TB or switched during treatment due to an incident adverse event)

We also identified factors that were associated with having a low PCS summary score Table 4. In the final adjusted multivariate regression both patient-reported AE (aRR 1.52 95% CI 1.07–2.18) and being on DR-TB treatment for less than 6 months (≤6 vs. > 6 months; aRR 1.70 95% CI 1.11–2.61) were associated with having a low PCS summary score.

## Discussion

Although other studies have reported that AEs reduce the overall quality of life of patients [38, 39], this is one of the largest studies to describe the impact of AEs on HRQoL among patients receiving treatment for DR-TB. Close to 40% of patients reported recently experiencing an AE, with joint pain, peripheral neuropathy, and hearing loss the most common [40]. In this cross-sectional study, we found that HRQoL, more specifically mental health and wellbeing, were lower among DR-TB patients who self-reported experiencing an AE. Not surprisingly, all eight domains and both summary scales (MCS and PCS) on the SF-36 were lower in patients receiving treatment for DR-TB compared to healthy adults.

In a context where 77.9% of patients are also infected with HIV, patients initiating ART are more likely to experience an AE in the first six month of DR-TB treatment [6]. We found that HRQoL was influenced by duration of treatment. Patients in the intensive phase (first 6 months) of DR-TB therapy were more likely to have a low MCS and PCS. Unexpectedly, just over half of the patients who reported AEs had been receiving DR-TB treatment for more than 6 months. This could signify that either the AEs in the latter portion of DR-TB treatment are not as severe as those in the intensive phase and hence have lesser effect on MCS and PCS, or that these are persistent AEs that are either better tolerated or better managed than they had been during the intensive phase of treatment.

The role of DR-TB in determining HRQoL of HIV co-infected patients remains unclear. When limiting our comparison to HIV-positive patients on ART, we discerned a lower MCS summary score among co-infected patients receiving treatment for DR-TB compared to patients with DS-TB or those without TB. Contrary to other reports we found that the MCS summary score was similar among HIV co-infected patients with DS-TB and those with HIV alone, possibly because all of the patients were receiving ART [41].

HIV co-infected patients receiving treatment for DR-TB, but not on ART, had a low MCS and PCS summary score compared to patients who were on ART. This is consistent with other reports demonstrating an improved quality of life following the start of ART [42]. Co-infected patients (DR- and DS-TB) had a low PCS summary score than those without TB, highlighting the need for strategies to incorporate social-psychological support and rehabilitation therapy to improve HRQoL. Some AEs (e.g. dizziness or vertigo, hearing loss and joint pain) are more prevalent in DR-TB patients co-infected with HIV [6], and those newly initiating ART. The persistence of AEs in HIV co-infected patients has been described [43] and may also possibly explain why patients continue to experience AEs throughout the course of DR-TB therapy.

In this study we show that patients on an individualized (injection-free) regimen had a better mental wellbeing, but similar PCS summary score, than those who were on a standard long- or short-course regimen containing kanamycin. In July 2018, South Africa became one of the first countries to recommend injection-free, BDQ regimen for all patients with RR-TB, including both short- and long-course regimens [25]. Two months later, the WHO announced a fully oral regimen as one of the preferred options for MDR-TB treatment, with injectable agents (e.g. kanamycin) proposed to be replaced by more potent alternatives such as BDQ (Fig. 1) [20]. In its recommendation, the WHO argued that evidence is currently lacking for an all-oral short-course regimen (i.e. bedaquiline, linezolid or delamanid replacing the injectable agent) as clinical trial data is not yet available. However, there is growing pressure for programs to eliminate injectable regimens [44], given the mounting evidence for the safety and efficacy of bedaquiline from observational studies [25] coupled with the known harm caused by injectable agents [6]. Our study contributes to the body of evidence demonstrating the negative impact of injectable agents on HRQoL. These negative effects of injectable agents on HRQoL, adherence and willingness to remain engaged in care need to be taken into account as countries decide to adopt oral or injectable regimens for RR/MDR-TB treatment in their national programs.

In our study we found that in most instances the MCS was lower than the PCS summary score. While this may reflect the positive effect of TB treatment on improving patients’ quality of life, as physical health tends to recover more quickly than mental well-being [12], it may also reveal the underlying driver of poor HRQoL. Untreated depression in patients with TB is associated with worse treatment outcomes, poor quality of life and greater disability [45]. Depression is often comorbid with TB and HIV and disproportionately affects those in lower socio-economic groups [46]. We note that a MCS lower than a PCS summary score must be interpreted with caution, however, as it may be an artefact of the way the summary scores are calculated [47]. Despite this, our results highlight the need to monitor mental health among DR-TB patients and provide support where possible. We recommend combining the SF-36 questionnaire with a screening tool for anxiety or psychological distress, such as the 10 item Kessler Psychological Distress Scale (K10) [48], throughout both the intensive and continuation phase of DR-TB treatment, to identify patients who require support or mental health care.

### Study limitations

Results should be considered in light of the study limitations. First, because this was a cross-sectional study, one of the major limitations is temporality. Since risk factors and outcomes are measured simultaneously, it was impossible to make inferences about causality. Another limitation of cross-sectional studies is the potential introduction of survival bias: serious AEs (e.g. hearing loss) or AEs with longer duration are sometimes overrepresented because patients are more likely to be included in the sample and are more likely to present with and report the AE compared to those who recover quickly (e.g. mild or moderate AE) or who die before they can be interviewed. Similarly, patients with potentially life threatening AEs often require hospitalization and would therefore have been excluded.

Second, for the norm-based approach, the 2016 United States population mean and standard deviation were used to standardize the score. Data for a South African reference population were not available. The standard scoring algorithm assumes that MCS and PCS summary scores are not correlated, which can sometimes result in inconsistent results between the SF-36 domains and the MCS or PCS summary scores [47]. For these reasons, we opted to present the normal additive approach (not standardized), the norm-based approach (standardized) and the MCS and PCS summary scores.

Third, majority of the participants were HIV positive and only 18.1% were HIV negative therefore no inferences could be made between the two groups. Furthermore, the comparison patient groups were small and were only included for comparative purposes.

Finally, HRQoL and AE data relied on patient self-report. AEs were not graded by the treating clinician (e.g. as mild/grade 1, moderate/grade 2, severe/grade 3, potentially life-threatening/grade 4 or fatal/grade 5). No laboratory values or clinical records were reviewed and AEs that occurred before the 4 week cut-off were excluded (except those that were persistent or caused disability e.g. hearing loss). Patients may have over- or under-reported symptoms or AEs during an interview. Questionnaires were administered in English and verbally translated into local languages by interviewers so a poor understanding of the questions may have contributed to reporting bias. To overcome this, we used a checklist system to capture the adverse drug reaction and also used open-ended questions to ask patients if they were having any problems [49]. To minimize bias, the interviewer trained to administer the questionnaire was not a TB clinic nurse and did not provide TB or HIV care to these patients. Studies have demonstrated a discordance between patient and clinician reports of AEs to MDR-TB treatment with underreporting of patient adverse drug reactions in medical records [50]. In future work we plan to explore the concordance/discordance between patient-reported and clinician documentation of AEs in this sample.

## Conclusion

Both DR-TB and HIV treatment pose the risk of AEs; concurrent treatment creates the potential for overlapping toxicities. Patients who reported an AE during DR-TB treatment experienced poorer HRQoL, affecting both their mental and physical health, than those who did not report an AE. We found that AEs contributed to poorer HRQoL during the first few months of treatment but diminished in impact on HRQoL as treatment continued. The availability of drugs with better safety profiles and management of patients that is more responsive (i.e. an integrative patient-centered approach) may result in improved HRQoL which will result in better treatment outcomes and contribute to global efforts to control TB [18].

## Additional files

Additional file 1: **Table S1.** Summary of approaches used to score the SF-36. (DOCX 16 kb)

Additional file 2: **Table S2.** Summary of symptoms and associated adverse events related to DR-TB treatment. (DOCX 24 kb)

Additional file 3: **Table S3.** Comparison of SF-36 health domain scales (norm-based) for healthy adults (n=40). (DOCX 15 kb)

## Abbreviations

AE: Adverse event
aHR: Adjusted hazard ratio
aRR: Adjusted relative risk
ART: Antiretroviral therapy
BDQ: Bedaqualine
COJ: City of Johannesburg
DR-: Drug resistant
DS-: Drug susceptible
HIV: Human immunodeficiency virus
HREC: Human research ethics committee – medical
HRQoL: Health-related quality of life
IQR: Interquartile range
LPA: Line probe assay
MCS: Mental component summary score
MDR-: Multi-drug resistant
MTB: *Mycobacterium tuberculosis*
NDOH: National Department of Health
NHLS: National Health Laboratory Service
PCS: Physical component summary score
PTB: Pulmonary tuberculosis
RIF: Rifampicin
RMR: Rifampicin mono-resistant tuberculosis
RR-TB: Rifampicin-resistant tuberculosis
TB: Tuberculosis
USA: United States of America
UTT: Universal test and treat
WHO: World Health Organization
XDR-: Extensively drug resistant
