# Supplementary material for: The impact of adverse events on health-related quality of life among patients receiving treatment for drug-resistant tuberculosis in Johannesburg, South Africa
Source: Health Qual Life Outcomes. 2019 May 31;17:94. doi: 10.1186/s12955-019-1155-4 (PMC6545023; doi:10.1186/s12955-019-1155-4)
Supplement: Supplementary file 1 — Table S1. Summary of approaches used to score the SF-36. (DOCX 16 kb) [file 12955_2019_1155_MOESM1_ESM.docx]

Supplementary Table 1. Summary of approaches used to score the SF-36.

| **Normal additive approach** |
| --- |
| - First, pre-coded numeric values were recoded according to the scoring key provided (e.g. the original response category in the questionnaire 1-5 was recoded as 100-0, in increments of 25). All items were scored so that a high score defined a more favorable health state. In addition, each item was scored on a 0 to 100 range so that the lowest and highest possible scores were 0 and 100, respectively. - Next, items were averaged together to create eight health concepts which included: physical functioning, bodily pain, role limitations due to physical health problems, role limitations due to personal or emotional problems, emotional well-being, social functioning, energy/fatigue, and general health perceptions. We also included a single item that provided an indication of a perceived change in health. - Items that were left blank (missing data) were not taken into account when calculating the domains so that scales represent the average for all items that the respondent answered. - The eight domains were aggregated into two summary measures: the physical (PCS) and mental (MCS) component summary scores. - We present information on the reliability using Cronbach’ alpha, central tendency, and variability of the domains scored using this method. A Cronbach’ alpha value of >0.80 was used to define good internal consistency of the SF-36 domains. |
| **Norm-based approach** |
| - We used norm-based transformation (also known as the norm-based approach) to standardize the observed score with reference to the population mean and standard deviation (SD) (Supina et al, 2006, Ware and Sherbourne, 1992). - First, the 0-100 scores for the eight subscales were standardized using a z-score transformation, which involves subtracting the population mean score for that scale from each respondent’s score, and dividing the difference by the population standard deviation. - Next, to give a mean of 50 and standard deviation of 10, the z-score is multiplied by 10 and 50 is added to the product (Norm-based score = [(observed score − population mean) ÷ population SD]×10+50). To produce the PCS and MCS summary scores, the z-scores for each of the eight domains was multiplied by a factor score coefficient (provided; reference the manual) and the resulting scores summed over the eight subscales. - Finally, the PCS and MCS summary scores are translated into t-scores (with a mean of 50 and a SD of 10) by multiplying the PCS and MCS summary scores by 10 and adding 50 to the product. |
