# Supplementary material for: The impact of adverse events on health-related quality of life among patients receiving treatment for drug-resistant tuberculosis in Johannesburg, South Africa
Source: Health Qual Life Outcomes. 2019 May 31;17:94. doi: 10.1186/s12955-019-1155-4 (PMC6545023; doi:10.1186/s12955-019-1155-4)
Supplement: Supplementary file 2 — Table S2. Summary of symptoms and associated adverse events related to DR-TB treatment. (DOCX 24 kb) [file 12955_2019_1155_MOESM2_ESM.docx]

Supplementary Table 2. Summary of symptoms and associated adverse events related to DR-TB treatment.

| **Adverse event** | **Symptoms** | **(n=122)** | **Standard long-course** | **Individualized**  **long-course** | **Standard short-course** | **Individualized short-course** |
| --- | --- | --- | --- | --- | --- | --- |
| **Visual disturbance** | Blurred vision | 4 | 2 | 1 | 1 | - |
|  | Double vision |  |  |  |  |  |
|  | Seeing less or poorer vision |  |  |  |  |  |
|  | Seeing (black) spots |  |  |  |  |  |
|  | Night blindness |  |  |  |  |  |
|  | Flashes of light |  |  |  |  |  |
|  | Painful eyes |  |  |  |  |  |
|  | Teary, watery eyes |  |  |  |  |  |
|  | Dry eyes |  |  |  |  |  |
|  | Burning, itchy or irritated eyes | |  |  |  |  |
|  | Inflamed eyes |  |  |  |  |  |
|  | Itchy or irritated eyelids |  |  |  |  |  |
|  | Inflamed eyelids |  |  |  |  |  |
|  | Puffy or swollen eyes or eyelids | |  |  |  |  |
|  | Enlarged pupils |  |  |  |  |  |
|  | Pressure on the eyes |  |  |  |  |  |
|  | Burst eye vessels |  |  |  |  |  |
|  | Inability to move eyes |  |  |  |  |  |
| **Anemia** |  | 1 | - | - | - | 1 |
| **Palpitations** | Palpitations | 1 | 1 | - | - | - |
|  | Rapid heartbeat |  |  |  |  |  |
|  | Slow heartbeat |  |  |  |  |  |
|  | Irregular heartbeat, arrhythmia | |  |  |  |  |
| **Nausea or vomiting** | Nauseous, sick | 12 | 6 | 2 | 3 | 1 |
|  | Acid indigestion, stomach acid, heartburn | |  |  |  |  |
|  | Vomiting reflex |  |  |  |  |  |
|  | Vomiting |  |  |  |  |  |
|  | Vomiting blood |  |  |  |  |  |
| **Abdominal pain** | Intestinal, stomach, abdominal cramps and/or pain | 2 | 2 | - | - | - |
| **Diarrhea** | Diarrhea | 2 | 1 | 1 |  |  |
|  | Runnier, softer feces (not diarrhea) | |  |  |  |  |
|  | Mucus in feces |  |  |  |  |  |
|  | More frequent bowel movements | |  |  |  |  |
| **Rash** | Painful skin | 5 | 4 | 1 | - | - |
|  | Itchiness |  |  |  |  |  |
|  | Blisters |  |  |  |  |  |
|  | Rashes (for example red patches, pimples) | |  |  |  |  |
|  | Spot (painful), ulcer, wound |  |  |  |  |  |
|  | Skin discoloration (for example yellow or pale skin) | |  |  |  |  |
| **Myalgia** | Muscle cramps (for example leg cramp) | 10 | 8 | 1 | 1 | - |
|  | Muscle pain, susceptible to muscle ache | |  |  |  |  |
|  | Muscle contractions |  |  |  |  |  |
|  | Tired, heavy muscles |  |  |  |  |  |
|  | Stiff muscles, stiffness (for example stiff neck) | |  |  |  |  |
|  | Quivering, trembling, shaking muscles | |  |  |  |  |
| **Joint pain** | Bone pain | 22 | 12 | 5 | 2 | 3 |
|  | Painful joints |  |  |  |  |  |
|  | Inflammatory arthritis (gout) |  |  |  |  |  |
|  | Stiff joints |  |  |  |  |  |
| **Peripheral neuropathy** | No or numb sensation | 16 | 8 | 4 | 2 | 2 |
|  | Tingling or prickling sensation | |  |  |  |  |
| **Dizziness or vertigo** | Unsteadiness, insecure, unsteady feeling | 11 | 5 | 3 | 3 | - |
|  | Balance problems |  |  |  |  |  |
|  | Falling |  |  |  |  |  |
|  | Fainting |  |  |  |  |  |
|  | Light-headedness |  |  |  |  |  |
|  | Dizziness |  |  |  |  |  |
| **Depression or mood disorder** | Lack of concentration | 9 | 7 | 2 | - | - |
|  | Over-sensitive, irritable |  |  |  |  |  |
|  | Restless |  |  |  |  |  |
|  | Aggressive |  |  |  |  |  |
|  | Nervous, tense |  |  |  |  |  |
|  | Anxious, fretful, worried |  |  |  |  |  |
|  | Lack of emotions |  |  |  |  |  |
|  | Over-emotional |  |  |  |  |  |
|  | Depressed, somber |  |  |  |  |  |
|  | Crying fits |  |  |  |  |  |
|  | Changed mood |  |  |  |  |  |
|  | Mood swings |  |  |  |  |  |
|  | Changed personality |  |  |  |  |  |
| **Psychosis** | Voices in the head | 2 | 1 | 1 | - | - |
|  | Hallucinations |  |  |  |  |  |
|  | Psychosis |  |  |  |  |  |
| **Headache** | Headache | 2 | 1 | - | 1 | - |
|  | Migraine |  |  |  |  |  |
| **Insomnia** | Sleep problems, sleeplessness | 2 | 2 | - | - | - |
|  | Dreams, nightmares |  |  |  |  |  |
| **Fatigue** | Fatigue | 6 | 5 | - | 1 | - |
|  | Listlessness, dullness, lethargy, lack of energy | |  |  |  |  |
| **Hearing loss** | Assessed using HHIE-S Hearing loss tool*  No handicap (HHIE-S score 0-8) n=3  Mild-moderate (HHIE-S score 9-24) n=7  Severe handicap (HHIE-S score 26-40) n=5 | 15 | 9 | 6 | - | - |

*Ventry and Weinstein, 1983

^a^ Standard long-course = 6 months of injectable kanamycin and 18-24 months of oral moxifloxacin, ethionamide, terizidone, and pyrazinamide.

^b^ Individualized long-course = bedaquiline was introduced as a substitute for kanamycin in the standard long-course regimen (either at start of DR-TB or switched during treatment due to an incident adverse event).

^c^ Standard short-course = 4 to 6-month intensive phase of kanamycin, moxifloxacin, ethionamide, clofazimine, pyrazinamide and high-dose isoniazid followed by 5 months of moxifloxacin, clofazimine, pyrazinamide and ethambutol.

^d^ Individualized short-course = bedaquiline was introduced as a substitute for kanamycin in the standard short-course regimen (either at start of DR-TB or switched during treatment due to an incident adverse event).
