# Supplementary material for: The impact of adverse events on health-related quality of life among patients receiving treatment for drug-resistant tuberculosis in Johannesburg, South Africa
Source: Health Qual Life Outcomes. 2019 May 31;17:94. doi: 10.1186/s12955-019-1155-4 (PMC6545023; doi:10.1186/s12955-019-1155-4)
Supplement: Supplementary file 3 — Table S3. Comparison of SF-36 health domain scales (norm-based) for healthy adults (n=40). (DOCX 15 kb) [file 12955_2019_1155_MOESM3_ESM.docx]

Supplementary Table 3. Comparison of SF-36 health domain scales (norm-based) for healthy adults (n=40)*

|  | **Healthy adults (n=40)^$^** | |
| --- | --- | --- |
| **SF-36 scale^$^  (Norm-based scale)** | **Mean** | **SD** |
| Physical functioning | 96.3 | 8.5 |
| Role functioning/physical | 90.6 | 23.8 |
| Role functioning/emotional | 81.2 | 32.2 |
| Energy/fatigue | 71.1 | 20.1 |
| Emotional well-being | 78.4 | 15.1 |
| Social functioning | 90.0 | 16.5 |
| Pain | 87.2 | 15.2 |
| General health | 83.8 | 16.3 |
|  |  |  |
| Mental health component summary (MCS) | 50.3 | 10.3 |
| Physical health component summary (PCS) | 57.6 | 5.1 |

*From published data (van Aswegen et al, 2011).
